# Self-Assembly Strategies in Upconversion Nanoparticle-Based Nanocomposites: Structure Designs and Applications

**DOI:** 10.3390/ijms26178671

**Published:** 2025-09-05

**Authors:** Zhen Zhang, Xiaoyu Ji, Weijia Huang, Qizhen Mai, Du Cheng

**Affiliations:** 1 PCFM Lab, Guangdong Engineering Technology Research Centre for Functional Biomaterials, School of Materials Science and Engineering, Sun Yat-sen University, Guangzhou 510275, China; 2 State Key Laboratory of Molecular Engineering of Polymers, Fudan University, Shanghai 200437, China; 3 State Key Laboratory of Advanced Polymer Materials, Sichuan University, Chengdu 610207, China

**Keywords:** self-assembly, upconversion, nanocomposites

## Abstract

Self-assembly has emerged as a powerful bottom-up strategy for the construction of multifunctional nanocomposites based on upconversion nanoparticles (UCNPs). In contrast to epitaxial shell growth, self-assembly enables the modular integration of UCNPs with a broad spectrum of other functional nanomaterials. This characteristic makes it particularly attractive for various practical applications. This review provides a comprehensive overview of self-assembly methodologies for UCNP-based nanocomposites, including electrostatic interactions, hydrophobic interactions, covalent coupling, and specific biorecognition. The resultant nanohybrids exhibit a wide range of morphologies and functionalities, making them suitable for various applications, including multimodal imaging, bioimaging, advanced biosensing, smart nanocarriers for controlled molecular delivery, and orthogonal photoactivation for programmable therapy. Key recent studies are highlighted to elucidate the structure–function relationships and technological potential of these materials. Additionally, the current challenges, such as stability, reproducibility, and functional integration, and proposed future directions for the development of UCNP-based nanocomposites are further discussed.

## 1. Introduction

Lanthanide-doped upconversion nanoparticles (UCNPs) have attracted significant attention due to their unique photophysical properties, such as anti-Stokes emission, sharp emission bands, long luminescence lifetimes, and superior photostability [1,2,3,4,5]. These features make UCNPs promising candidates for diverse applications in bioimaging, phototherapy, optoelectronics, and security encoding [6,7,8,9,10]. Nevertheless, their practical performance is often limited by low excitation efficiency, narrow absorption profiles, and insufficient emission tunability. To overcome these drawbacks, recent strategies have focused on fabricating hybrid nanostructures by integrating UCNPs with complementary functional materials [11,12,13,14]. Conventional core–shell epitaxial growth methods have enabled the formation of structurally coherent heteronanostructures with improved energy transfer and surface passivation [15,16,17,18]. However, these approaches usually require high-temperature conditions and lattice-matching constraints, which restrict the choice of hybrid components and limit process scalability. Moreover, such synthetic routes are incompatible with biologically active molecules or temperature-sensitive materials. Self-assembly offers a flexible and scalable alternative for engineering UCNP-based nanocomposites [19,20,21,22,23]. This bottom-up approach leverages a variety of non-covalent and covalent interactions to assemble UCNPs with other nanomaterials in an ambient or aqueous environment. By tailoring surface chemistry, solvent polarity, and external stimuli, a diverse range of architectures can be constructed with high precision and modularity. Importantly, self-assembly enables the incorporation of soft materials, stimuli-responsive polymers, and biomolecules, thereby expanding the functional landscape of UCNPs.

In this review, the principal self-assembly mechanisms utilized in the synthesis of UCNP-based nanocomposites are discussed. For each mechanism, we highlight representative studies and emphasize how the interaction types influence the composite morphology and performance. Applications in key areas include multimodal imaging, bioimaging, advanced biosensing, smart nanocarriers for controlled molecular delivery, and orthogonal photoactivation for programmable therapy. Finally, we outline the current limitations and suggest future research perspectives to guide the rational design of advanced UCNP-based nanocomposites.

## 2. Self-Assembly Mechanisms for UCNP-Based Nanocomposites

### 2.1. Electrostatic Interactions

**Figure 1:**
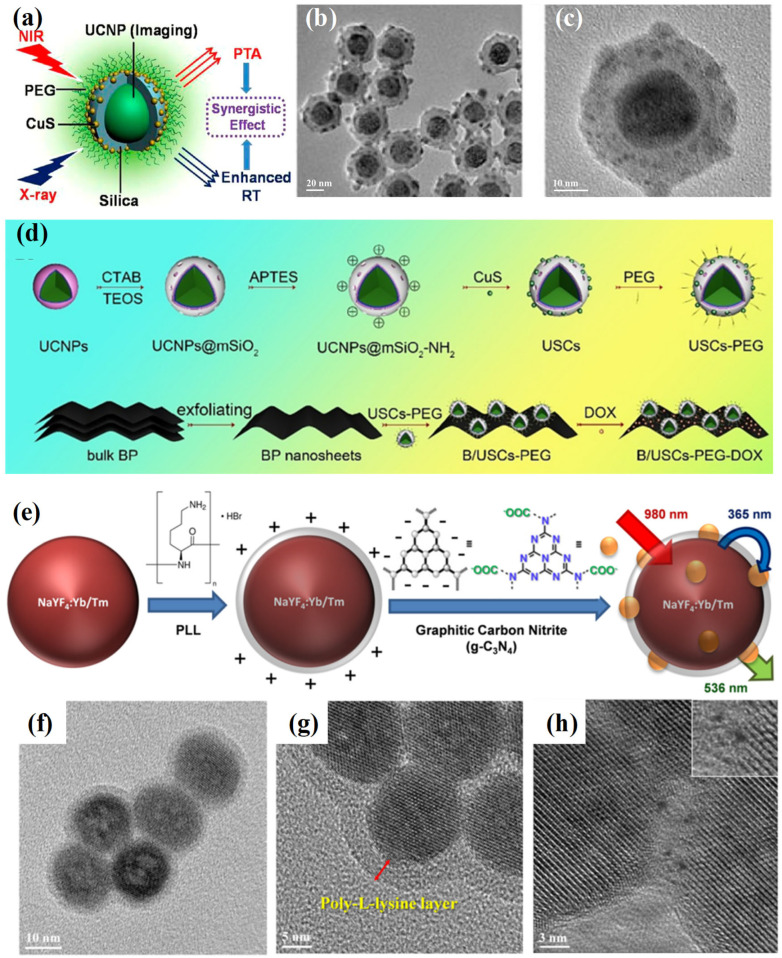
(**a**) Schematic illustration of UCNPs/CuS core–satellite nanostructures designed for combined radiotherapy and photothermal therapy. UCNPs enhance local radiation dose, while CuS nanoparticles enable 980 nm laser-induced heat generation. (**b**,**c**) TEM images of the assembled UCNPs/CuS core–satellite nanostructures [24]. (**d**) Synthetic route of B/USCs-PEG-DOX nanoplatform [25]. (**e**) Stepwise synthesis of UCNPs-PLL@g-C_3_N_4_ hybrid. (**f**) TEM image of UCNPs coated with PLL. (**g**) Enlarged view showing the polymer layer on UCNPs. (**h**) Deposition of g-C_3_N_4_ on the PLL-coated surface (inset: zoomed-in region of g-C_3_N_4_ attachment) [29].

Electrostatic interactions represent one of the most accessible and widely applied mechanisms for constructing UCNP-based nanocomposites. By tailoring the surface charge of UCNPs and their counterpart materials, electrostatic attraction can drive the formation of nanocomposites under mild, aqueous conditions without the need for harsh solvents or elevated temperatures. UCNPs are typically modified with charged functional groups to enable such interactions [24,25,26,27,28]. For instance, positively charged amine functionalities can be introduced via surface grafting with (3-aminopropyl) triethoxysilane (APTES) or poly (ethyleneimine) (PEI), while negatively charged surfaces are commonly achieved using carboxyl or phosphate terminal groups. These modifications permit the electrostatic binding of complementary nanocomponents, including metal sulfide nanoparticles, two-dimensional nanosheets, and metal–organic frameworks (MOFs). A representative example involves the construction of UCNPs@CuS composites through charge-guided assembly (Figure 1a–c) [24]. Positively charged UCNP@SiO_2_-NH_2_ particles readily adsorb negatively charged CuS nanoparticles, yielding a core–satellite structure that demonstrates enhanced photothermal conversion under near-infrared (NIR) illumination. This charge-mediated coupling not only simplifies fabrication but also preserves nanoscale dispersion in physiological environments. Complex multi-component assemblies can also be realized using sequential electrostatic binding. In one study, amino-functionalized UCNPs@mSiO_2_ particles were first combined with CuS nanoparticles, followed by PEGylation to improve colloidal stability, and finally hybridized with negatively charged black phosphorus (BP) nanosheets (Figure 1d) [25]. The resulting construct effectively merged disparate components into a cohesive nanoplatform with synergistic functionalities. Electrostatic interactions have further enabled the integration of UCNPs with functional polymers and 2D semiconductors. For example, g-C_3_N_4_ sheets with carboxyl-rich surfaces have been successfully interfaced with UCNPs bearing poly (L-lysine) coatings, yielding nanohybrids capable of responsive fluorescence emission (Figure 1e–h) [29].

**Figure 2:**
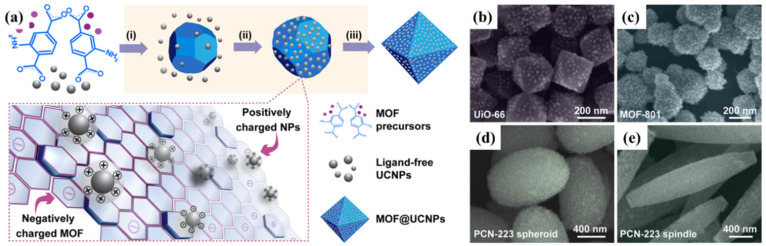
(**a**) Schematic depicting the assembly of MOF-UCNPs nanocomposites via direct mixing of MOF precursors and ligand-free UCNPs. The shaded sequence illustrates the proposed mechanism: (i) MOF nucleation, (ii) electrostatic attachment of UCNPs, and (iii) nanocomposite formation. Here, MOF@UCNPs refers to the resulting hybrid structures. (**b**–**e**) SEM images of representative composites: UiO-66@UCNPs (**b**), MOF-801@UCNPs (**c**), and PCN-223@UCNPs (**d**,**e**) [34].

In addition, porous materials such as MOFs have been decorated with UCNPs by leveraging charge complementarity [30,31,32,33,34,35]. Frameworks including UiO-66, MOF-801, and PCN-223 have served as scaffolds for electrostatic adsorption of lanthanide-doped nanoparticles, expanding the utility of UCNPs in catalysis and molecular recognition (Figure 2) [34].

The versatility of electrostatic assembly lies in its sensitivity to the physicochemical environment. Parameters such as pH, ionic strength, and surface ligand density can be tuned to modulate interparticle distances, assembly rates, and overall architecture, ranging from core-shell structures to layered films or densely packed aggregates. Nonetheless, high salt concentrations or serum proteins may compromise assembly stability by screening surface charges or promoting nonspecific aggregation. To mitigate these effects, zwitterionic stabilizers or charge-balanced coatings are increasingly adopted to enhance dispersibility under biologically relevant conditions. Taken together, electrostatic self-assembly offers a facile, tunable, and modular route for constructing UCNP-based hybrid nanostructures, with demonstrated applications in photothermal therapy, bioimaging, and sensing. Future advancements may include multi-channel electrostatic coding for orthogonal component integration and adaptive interface engineering to resist biofouling in complex environments

### 2.2. Hydrophobic Interaction

**Figure 3:**
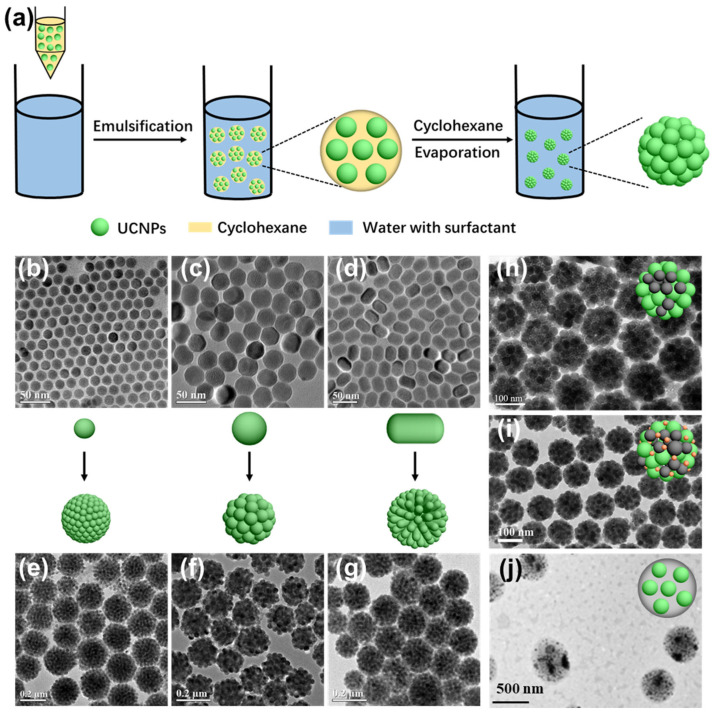
(**a**) Schematic representation of UCNP cluster formation via a microemulsion-assisted approach [4]. (**b**–**d**) TEM images of UCNPs with distinct shapes and sizes: spherical (**b**,**c**) and rod-like (**d**). (**e**–**g**) Corresponding assembled clusters derived from the UCNPs in (**b**–**d**) [37]. TEM images of UCNP-based hybrid nanostructures: (**h**), UCNP/Fe_3_O_4_ composites [43]; (**i**), ternary UCNP/Fe_3_O_4_/Au hybrids [44]; (**j**), PLGA-associated UCNP assemblies [46].

Hydrophobic interactions play a pivotal role in guiding the self-organization of UCNPs, particularly when the particle surface is functionalized with nonpolar ligands such as oleic acid or embedded within amphiphilic environments. These interactions arise from the system’s thermodynamic drive to minimize unfavorable contact between hydrophobic surfaces and aqueous surroundings, leading to the spontaneous formation of organized nanostructures. Among several available assembly strategies, microemulsion templating and polymer-mediated encapsulation are two of the most effective techniques that leverage this principle. In microemulsion-assisted assembly, stable nanodroplets are formed in biphasic environments, commonly water-in-oil or oil-in-water systems, by surfactants such as CTAB, SDS, or nonionic agents such as Tween 20 [36,37,38,39,40,41]. Hydrophobically capped UCNPs tend to partition within the hydrophobic interior of these nanodroplets. Upon gradual low-evaporation boiling solvent removal, these UCNPs aggregate into uniform three-dimensional superstructures, typically exhibiting spherical morphologies (Figure 3a) [4]. For example, Bai et al. demonstrated that this approach enables the formation of dense, monodisperse UCNP clusters through a one-step microemulsion route [42]. Zhang and colleagues extended this method to systematically assemble UCNPs with varying morphologies, including spherical particles (~17 nm and ~34 nm in diameter) and nanorods, into compact colloidal spheres, confirming the broad applicability of this technique (Figure 3b–g) [37]. Furthermore, this method allows for the facile integration of heterogeneous nanoparticles within the same structure. For instance, Liu et al. successfully co-assembled UCNPs with Fe_3_O_4_ to fabricate dual-modal magnetic-luminescent superparticles (Figure 3h) [43]. Expanding on this, Liang et al. further incorporated Au nanoparticles into the existing UCNP/Fe_3_O_4_ framework to yield ternary multifunctional constructs (Figure 3i) [44], providing enhanced photothermal and imaging functionalities. The modularity of the microemulsion method also enables hybrid assemblies containing both inorganic and organic components. Dai’s group employed an amphiphilic polymer, PMAO-PEG, to encapsulate Zn-phthalocyanine (ZnPc) and UCNPs into composite nanoclusters via spontaneous assembly through this microemulsion method [45]. Similarly, Zhao et al. constructed pH-sensitive nanocapsules by co-assembling PLGA with UCNPs (Figure 3j), enabling the smart release of doxorubicin (DOX) in acidic tumor microenvironments [46].

**Figure 4:**
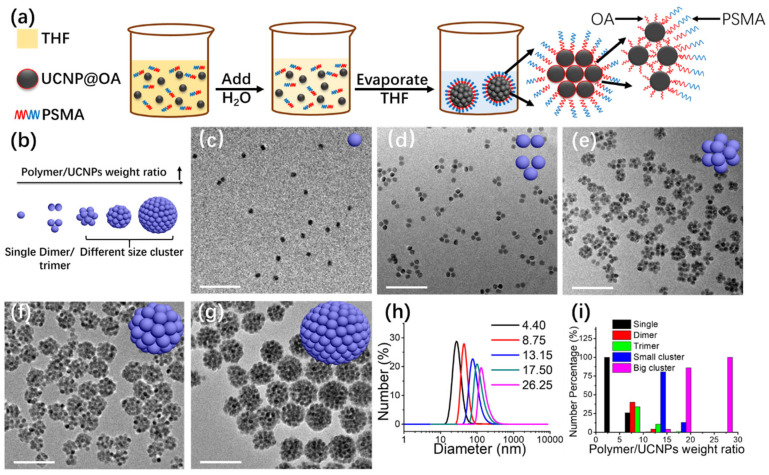
(**a**) Schematic illustration of PSMA-directed UCNP assembly. (**b**) Diagram showing the influence of UCNP-to-polymer weight ratios on assembly morphology. (**c**–**g**) TEM images of UCNP assemblies formed at weight ratios of 4.40, 8.75, 13.15, 17.50, and 26.25, respectively (scale bar: 200 nm). (**h**) DLS analysis of a representative UCNP assembly. (**i**) Distribution percentages of distinct assembly types under varying UCNP/polymer ratios [37].

The microemulsion templating technique represents a commonly employed strategy for assembling UCNPs into spherical superstructures from well-dispersed individual nanoparticles. The final cluster dimensions are influenced by parameters such as the relative proportions of organic solvents, aqueous phase, particle concentration, and surfactant content [47,48,49]. However, due to the intricate multiphase equilibria in these systems, achieving precise and uniform control over particle size and size distribution remains a considerable challenge. Recent advances suggest that polymer-mediated strategies offer enhanced control over nanoparticle organization [50,51,52,53,54,55,56,57,58]. Amphiphilic block copolymers have been shown to facilitate the dispersion of hydrophobic UCNPs in water by forming micellar structures. In these assemblies, the hydrophobic segments associate with the nanoparticle surface, sequestering them within the micelle core, while the hydrophilic portions extend outward, promoting colloidal stability. This self-assembly approach allows the tuning of micelle properties, including overall size, internal composition, and surface functionality. Additionally, the micellar framework readily accommodates the co-encapsulation of various hydrophobic molecules, such as therapeutic drugs or fluorophores, creating versatile nanoplatforms suitable for imaging, synergistic therapy, and integrated diagnostic applications. For example, Zhang’s team employed poly (styrene-*co*-maleic anhydride) (PSMA) to guide the formation of UCNP-based spherical assemblies with tunable geometries ranging from monomers and dimers to large clusters (Figure 4a) [37]. Their method involved mixing a PSMA solution in THF with UCNPs dispersed in cyclohexane, followed by gradual water addition. As THF evaporated at room temperature, the hydrophobic oleic acid ligands on UCNPs prompted aggregation to minimize exposure to water. Concurrently, the amphiphilic PSMA chains associated with the UCNP surfaces, forming hydrophilic shells that improved aqueous dispersibility. Upon further solvent removal, PSMA-PSMA interactions became dominant, driving tighter aggregation and the formation of larger clusters with reduced water contact area (Figure 4b–i). This approach enabled precise control over assembly size by adjusting the polymer-to-particle ratio and was later extended to anisotropic UCNPs, such as nanorods.

**Figure 5:**
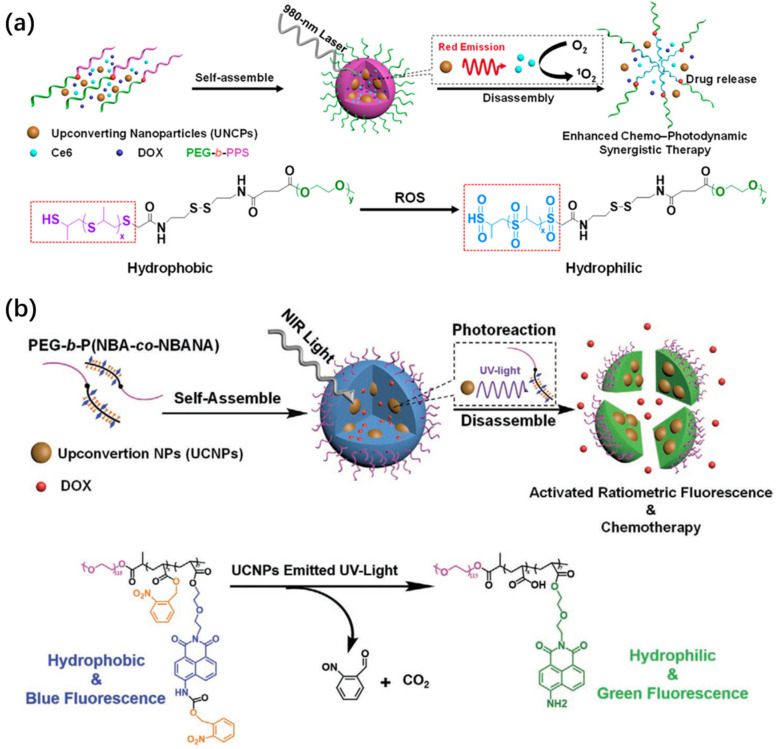
(**a**) Schematic illustration of the construction of ROS-responsive hybrid micelles co-loaded with Ce6 and DOX using UCNPs and polymer carriers [65]. (**b**) Upon UV emission from UCNPs, PEG-*b*-P (NBA-*co*-NBANA) undergoes a photo-triggered transformation from hydrophobic to hydrophilic, resulting in micelle disruption and DOX release [66].

Stimuli-responsive polymeric nanostructures have attracted increasing interest for their ability to respond to intracellular triggers such as enzymes, ROS, and redox-active agents such as glutathione [59,60,61,62,63,64]. These endogenous signals can be harnessed to initiate drug release, disrupt nanostructures, or enhance imaging contrast. Based on this concept, UCNP–polymer hybrid micelles have been developed for cancer therapy and cellular diagnostics. Zhu et al. designed a ROS-sensitive micellar platform by integrating UCNPs with PEG-*b*-PPS block copolymers (Figure 5a) [65]. In this system, the hydrophobic PPS domains are anchored onto oleic acid-coated UCNPs to drive self-assembly, while PEG ensure aqueous stability. Therapeutic cargos DOX and chlorin e6 (Ce6) were co-loaded via nanoprecipitation. Under 980 nm excitation, the UCNPs emitted at ~650 nm, which aligns well with Ce6′s absorption and boosts ROS generation. The resulting oxidative cleavage transformed PPS into hydrophilic fragments, destabilizing the micelles and triggering DOX release. The team also developed a dual-functional micelle composed of UCNPs and amphiphilic copolymers bearing naphthalimide fluorophores and 2-nitrobenzyl photocages (Figure 5b) [66]. Upon NIR-induced UV emission from UCNPs, photocleavage released charged functional groups, simultaneously disrupting micelle integrity and modulating fluorescence output. Through careful selection of polymer chemistry and upconversion emission characteristics, such nanoplatforms can be engineered for responsive drug release, spectral tuning, and real-time imaging in complex biological environments.

### 2.3. Covalent Coupling

**Figure 6:**
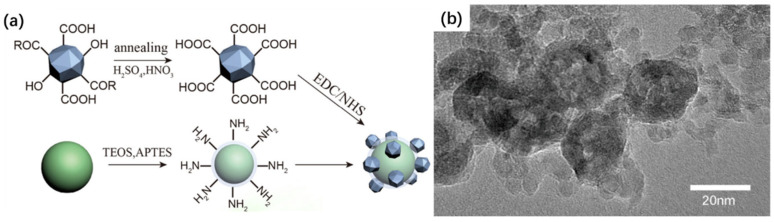
(**a**) Schematic representation of the fabrication and application of the UCNP–nanodiamond hybrid system. (**b**) TEM image of the resulting UCNP–nanodiamond composite structure [67].

Covalent self-assembly provides a highly stable and irreversible means to engineer UCNP hybrids with enhanced mechanical and chemical robustness. Unlike physical interactions, such as electrostatic attraction or hydrogen bonding, covalent coupling yields strong interfacial linkages that are resistant to disruption by dilution, ionic changes, or enzymatic degradation, making it ideal for in vivo biomedical applications and long-term storage [67,68,69,70]. Several well-established chemistries underpin this approach. Among them, carbodiimide-mediated amide coupling is widely used, where 1-ethyl-3-(3-dimethylaminopropyl)carbodiimide (EDC), often in combination with N-hydroxysuccinimide (NHS), activates carboxyl groups to react with primary amines, forming stable amide bonds [71,72,73]. Additionally, silanization reactions using alkoxysilanes (e.g., APTES) are effective for modifying UCNP surfaces with organosilane layers, providing versatile anchor points for subsequent bioconjugation [74,75]. Click chemistry and thiol-ene additions are also increasingly employed for bio-orthogonal and selective coupling under mild conditions [76,77,78]. This covalent strategy is widely adopted to tether UCNPs to a diverse range of functional nanomaterials, facilitating the fabrication of hybrid platforms with precise control over spatial organization and stoichiometry. For instance, ZnO-gated UCNP@mSiO_2_ assemblies have been synthesized by conjugating amine-functionalized ZnO nanoparticles to carboxyl-modified UCNP@mSiO_2_ using EDC-mediated crosslinking, thereby enabling pH-responsive drug release and upconversion-triggered gating [70]. In another example, nanodiamonds bearing surface carboxyl groups were covalently linked to amino-functionalized UCNPs to produce multifunctional constructs with enhanced photostability and cellular compatibility (Figure 6) [67].

Compared to non-covalent methods, covalent coupling offers several distinct advantages: enhanced durability in complex biological or catalytic environments, improved reproducibility and storage stability for clinical or industrial deployment, and scalable multilayer architecture, enabling stepwise functionalization and complex hierarchical assemblies. Meanwhile, dynamic covalent chemistry offers reversible yet robust linkages that combine the structural stability of covalent bonds with the adaptability of non-covalent interactions, thus enabling error-correction, recyclability, and stimuli-responsive behavior in UCNP assemblies. Taken together, covalent assembly routes expand the versatility of UCNP-based composites, supporting their application in environments where reliability and performance under physiological stress are critical.

### 2.4. Biorecognition-Mediated Assembly

Self-assembly driven by biorecognition interactions offers unparalleled selectivity and dynamic control, making it highly suitable for biomedical applications. These interactions, derived from natural molecular affinity systems, enable modular and programmable integration of functional components. Among commonly used recognition systems are biotin–streptavidin coupling [79], antigen–antibody interactions [80], and sequence-specific nucleic acid hybridization [81,82]. These molecular tools allow UCNPs to be precisely organized into hierarchical nanostructures with high reproducibility and biological responsiveness.

**Figure 7:**
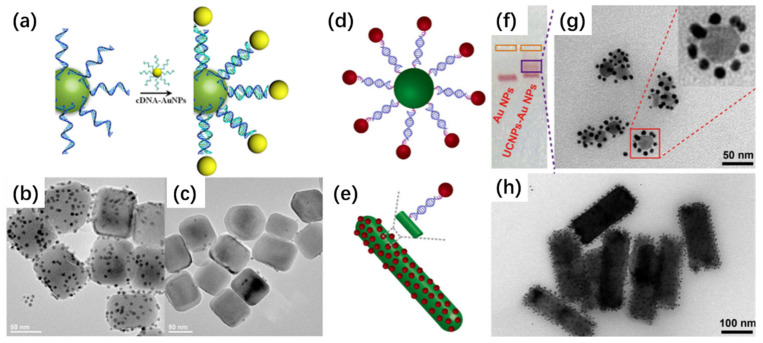
(**a**) Illustration of DNA-mediated hybridization between UCNPs and AuNPs. (**b**) TEM image of assembled nanostructures formed via specific binding between T30-modified UCNPs and complementary DNA-functionalized AuNPs. (**c**) The control sample with non-matching sequences shows no assembly, confirming sequence specificity [83]. (**d**,**e**) Schematic of programmable co-assembly of spherical and rod-shaped UCNPs with 5 nm AuNPs via DNA linkers. (**f**–**h**) TEM validation of the corresponding nanocomposites, demonstrating sequence-directed organization [84].

DNA has proven to be a versatile and programmable scaffold for nanoparticles assembly. By leveraging its predictable Watson–Crick base pairing and sequence addressability, researchers have constructed sophisticated architectures with spatial precision. In one representative example, UCNPs functionalized with thymine-rich oligonucleotides (e.g., T30) were hybridized with gold nanoparticles (Au NPs) bearing complementary adenine-rich sequences (e.g., A27), resulting in a satellite-like configuration where AuNPs surround a central UCNP core (Figure 7a–c) [83]. Control experiments using non-complementary sequences confirmed that hybridization was essential for the formation of these nanostructures, demonstrating the specificity of the DNA-mediated recognition. Further refinements to this strategy were reported through the use of monodisperse DNA-modified UCNPs and Au NPs to generate well-defined assemblies with tunable interparticle distances. By tailoring the DNA spacer length and sequence, hybrid nanostructures with precise control over nanoscale spatial arrangement were achieved. Such control enables the modulation of interparticle Förster resonance energy transfer (FRET), providing a sensitive platform for ratiometric sensing and molecular logic gate construction (Figure 7d–h) [84]. In a more complex application, DNA frameworks were employed to orchestrate the assembly of multicomponent nanopyramid structures composed of UCNPs, Cu_9_S_5_, Ag_2_S, and AuNPs. Upon the introduction of specific microRNAs, competitive hybridization induced the dissociation of recognition strands from the scaffold, resulting in the activation of two distinct luminescence signals in the visible and NIR-II regions [85]. This system demonstrates how biorecognition can be exploited not only for structural construction but also for dynamic response to biological stimuli. Recent advances in DNA nanotechnology, particularly DNA-origami, have established it as a powerful tool for constructing UCNP assemblies with nanometer-scale precision. By exploiting the programmable nature of DNA base-pairing, UCNPs can be positioned at predefined sites within a DNA scaffold, enabling highly controlled spatial arrangements and programmable lattice formation. This degree of control far surpasses that of conventional self-assembly methods, where interparticle spacing and orientation are often governed by nonspecific interactions. A key advantage of DNA-origami-mediated strategies is their ability to generate ordered superlattices with tunable interparticle distances, which directly affect optical coupling phenomena such as FRET, plasmon–exciton interactions, and collective emission effects. These programmable architectures not only provide a versatile platform to probe fundamental aspects of nanoscale photophysics but also enable the rational design of UCNP-based nanocomposites with tailored functional outputs [86,87,88,89].

Beyond DNA, antibody-functionalized UCNPs have been widely used to target specific biomarkers, enabling selective imaging of diseased cells or tissues. The use of aptamers and peptide ligands further expands the toolbox for biorecognition-guided assembly, offering diverse routes to target-specific functionality. In summary, molecular recognition-based assembly pathways provide a robust foundation for the rational design of UCNP-based nanocomposites with tunable architecture and functionality. They are particularly promising for constructing biosensors, programmable imaging agents, and responsive therapeutic platforms capable of operating in complex biological environments.

The nature of self-assembly forces plays a decisive role in defining the interfacial properties of UCNP-based nanocomposites. Electrostatic interactions can precisely tune interparticle spacing and facilitate controlled energy transfer pathways, directly impacting photoluminescence efficiency and FRET dynamics. Hydrogen bonding and van der Waals forces, though generally weaker, often lead to flexible and reversible assemblies that promote adaptive optical responses and dynamic sensing functionalities. By contrast, coordination interactions (e.g., metal–ligand binding) provide strong and directional linkages that enhance electronic coupling across interfaces, thereby improving charge-transfer efficiency and catalytic reactivity. Hydrophobic interactions can drive phase-segregated assemblies, enabling anisotropic architectures that reshape light scattering and emission profiles. Collectively, the interplay of these forces not only dictates interfacial geometry but also governs key functional outcomes, including quantum yield, upconversion efficiency, charge separation, and reaction kinetics. A systematic understanding of how specific self-assembly forces correlate with interfacial optical and catalytic properties is therefore essential for the rational design of UCNP nanohybrids optimized for advanced applications in imaging, sensing, and photocatalysis. To consolidate the above discussion, we provide a comparative overview of the main self-assembly strategies. A critical comparison is essential to understand how different interaction forces govern the structural and functional outcomes of UCNP-based nanocomposites. Four representative routes, electrostatic interactions, hydrophobic interactions, covalent coupling, and biorecognition-mediated assembly, capture the fundamental principles underlying most reported systems. Each relies on distinct physicochemical driving forces and offers unique control over interparticle spacing, structural rigidity, and interfacial stability, which ultimately determine optical performance, colloidal robustness, and biocompatibility. Table 1 summarizes these approaches side by side, outlining their operating conditions, structural features, optical outcomes, stability, and translational relevance. This framework highlights both the complementary strengths and inherent limitations of each method, while pointing to hybrid or integrative strategies as promising directions for advanced biomedical and photonic applications.

## 3. Applications of Self-Assembled UCNP Composites

The self-assembly of UCNPs into ordered and functional architectures has led to a broad array of biomedical and theranostic applications. These assemblies not only enhance the physicochemical and optical properties of individual UCNPs but also provide novel design platforms for multi-functionality. Recent advances include their application in multimodal imaging, bioimaging, advanced biosensing, smart nanocarriers for controlled molecular delivery, and orthogonal photoactivation for programmable therapy. This section summarizes the state-of-the-art in these fields, emphasizing how the rational design of UCNPs assemblies enables programmable and synergistic biomedical functionalities.

### 3.1. Multimodal Imaging

**Figure 8:**
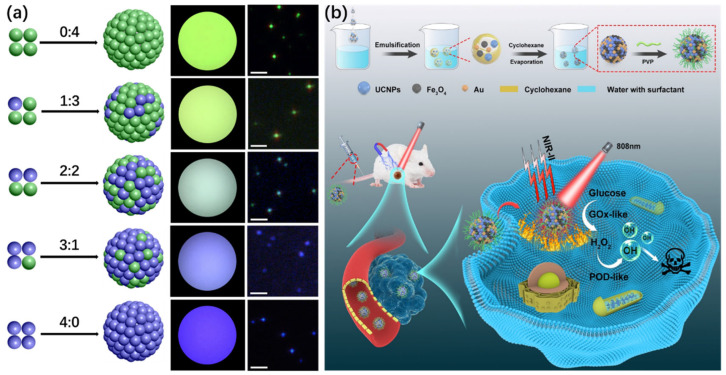
Schematic illustrating fluorescence color encoding through modulation of blue-to-green emission ratios (**a**) [37] and the microemulsion-based synthesis of UCNPs/Au/Fe_3_O_4_ hybrid constructs designed for NIR-II-guided, magnetically targeted, and photothermally enhanced catalytic cancer therapy (**b**) [44]. Scale bar in (**a**): 2 µm.

Due to their unique optical characteristics, such as high tissue penetration, photostability, and low autofluorescence under NIR excitation, UCNPs are highly suitable for in vivo imaging [5,90,91,92,93,94]. The rapid progress of NIR-II (1000–1700 nm) excitation/emission UCNP systems has created new opportunities in biomedical imaging. In comparison with conventional visible or NIR-I modalities, NIR-II platforms provide substantially deeper tissue penetration, lower background autofluorescence, and improved imaging contrast. These advancements have significantly expanded the utility of UCNP-based probes for in vivo bioimaging, particularly in deep-tissue applications, and mark a critical step toward clinical translation [95,96,97,98,99,100,101,102]. What is more, their self-assembled forms further allow integration with QDs, Au NPs, and fluorescent dyes, enabling multimodal and multiplexed imaging modalities. For example, microemulsion-based strategies allow UCNPs with distinct emission profiles to co-assemble into hybrid clusters, facilitating spectral encoding without complex structural engineering. By varying the ratios of blue- and green-emitting UCNPs under 980 nm excitation, clusters exhibiting tunable fluorescence at 450 nm and 550 nm were fabricated (Figure 8a) [37]. Additionally, multifunctional composites incorporating UCNPs, Fe_3_O_4_, and Au NPs have been developed for NIR-II fluorescence-guided cancer theranostics, offering real-time visualization of nanocarrier distribution and therapeutic response with high signal-to-noise ratios (Figure 8b) [44].

**Figure 9:**
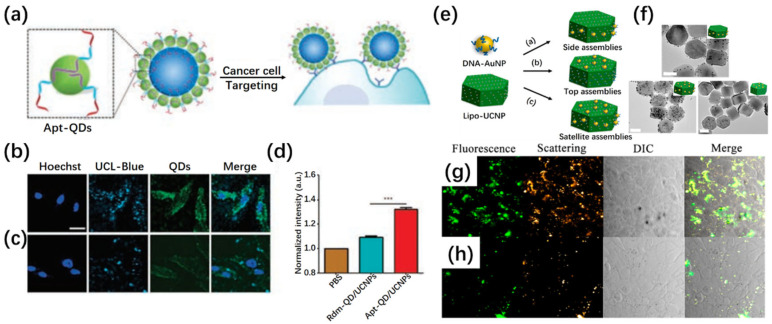
(**a**) Schematic illustration of Apt-UCNP/QD assemblies designed for targeted imaging of cancer cells. (**b**,**c**) Confocal images of MDA-MB-231 cells incubated with aptamer-functionalized (**b**) and random DNA-modified (**c**) UCNP/QD assemblies. Scale bar: 20 µm. (**d**) Flow cytometry analysis quantifying cellular uptake of both constructs [103]. *** *p* < 0.001. (**e**) Conceptual diagram of lab-on-a-particle hetero-assemblies and (**f**) corresponding TEM micrograph. Scale bar in (**f**): 50 nm (**g**,**h**) Confocal images of 4T1 cells treated with satellite assemblies bearing (**g**) specific aptamers or (**h**) non-targeting control strands, demonstrating dual-modality imaging capability [104].

DNA-guided self-assembly has also been leveraged to create UCNP/QD hybrid nanoarchitectures with enhanced dual-emission characteristics. In one design, CdTe QDs were templated with DNA oligonucleotides and selectively anchored to the periphery of UCNPs through strong coordination with surface lanthanide ions (Figure 9a–d) [103]. This satellite-like construct preserved the distinct emission properties of both components while facilitating cancer cell targeting via aptamer functionalization. Confocal microscopy and flow cytometry confirmed their effective intracellular localization and specific cellular uptake, highlighting their potential for integrated upconversion and fluorescence imaging. Similarly, anisotropic UCNP surfaces have been employed to spatially control the assembly of plasmonic AuNPs via sequence-specific DNA hybridization (Figure 9e–h) [104]. The resulting UCNP/Au NP satellite structures offered simultaneous plasmonic scattering and upconversion luminescence, enabling real-time visualization of biomolecular interactions and cellular environments. When conjugated with nucleolin-targeting aptamers, these assemblies provided high-contrast imaging of cancer cells, outperforming control assemblies with scrambled sequences.

### 3.2. Advanced Biosensing

**Figure 10:**
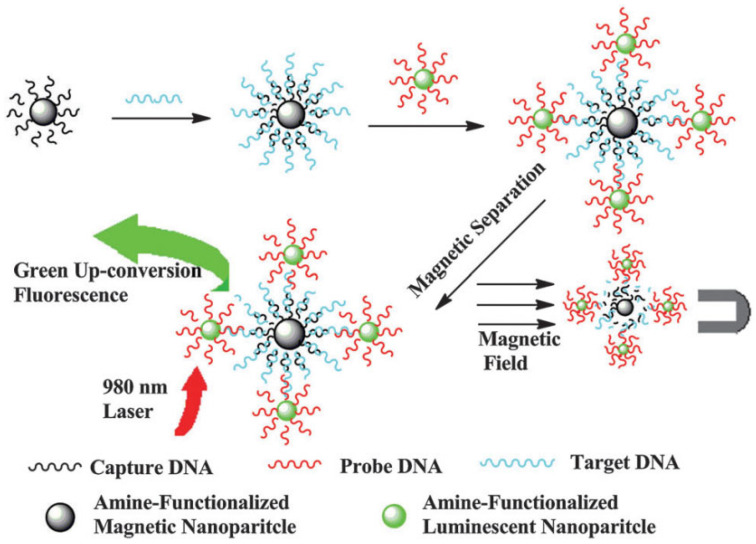
UCNPs functionalized with probe DNA hybridize with complementary sequences on magnetic nanoparticles, enabling the selective isolation of the complexes under an external magnetic field [108].

UCNPs have also gained traction in biosensing, particularly due to their ability to transduce biological recognition events into optical signals in NIR-transparent windows. Their upconversion emission is minimally affected by autofluorescence or photobleaching, thus enabling robust detection platforms [105,106,107]. To amplify signal intensity and target selectivity, researchers have developed heterostructured constructs by integrating UCNPs with magnetic Fe_3_O_4_ NPs or molecular beacons (Figure 10) [108]. In a DNA hybridization assay, UCNPs and Fe_3_O_4_ NPs were linked through complementary sequences, allowing magnetic separation of hybridized complexes and enabling sensitive luminescence detection of nucleic acids. Such sandwich-type biosensors demonstrate high specificity and fast signal readout, which are essential for molecular diagnostics. A recent study reported a luminescence resonance energy transfer (LRET)-based biosensing platform that integrates UCNPs with Au NPs for highly sensitive viral cDNA detection. Using SARS-CoV-2 cDNA as a model, UCNPs and Au NPs were functionalized with complementary primers to enable sequence-specific hybridization, achieving a detection limit of 242 fM. The system exhibited strong quenching efficiency, precise mismatch discrimination, and high reproducibility, demonstrating its promise for rapid and reliable viral diagnostics. Beyond COVID-19, this approach is adaptable for detecting diverse pathogens, offering a versatile route toward next-generation quantum-enhanced biosensors [109].

### 3.3. Smart Nanocarriers for Controlled Molecular Delivery

**Figure 11:**
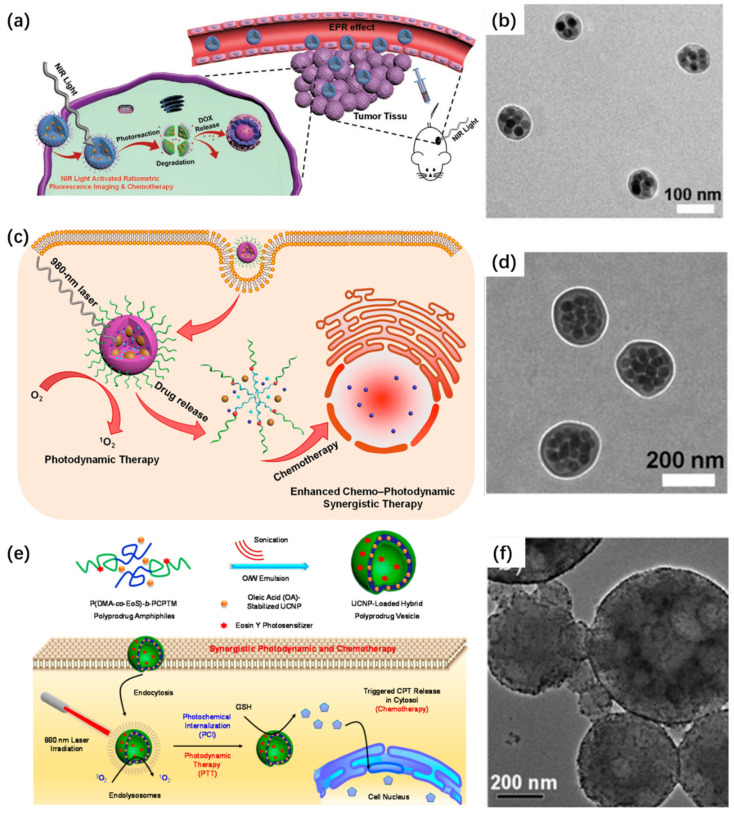
(**a**) NIR-triggered ratiometric fluorescence imaging and chemotherapeutic activation within cells. (**b**) TEM image of UCNP–polymer hybrid micelles [66]. (**c**) Intracellular synergistic chemo-photodynamic therapy (chemo-PDT). (**d**) TEM of hybrid micelles related to (**c**) [65]. (**e**) Schematic of UCNP-loaded vesicle fabrication using P (DMA-*co*-EoS)-*b*-PCPTM diblock copolymers via oil-in-water emulsification and solvent evaporation. (**f**) TEM image of polymeric vesicles containing UCNPs, corresponding to (**e**) [110].

The assembly of UCNPs into cluster-like or core–shell morphologies has opened avenues for the design of intelligent nanocarriers capable of stimuli-triggered release. Compared with conventional drug-loading strategies (e.g., physical adsorption or covalent conjugation), self-assembled architectures provide internal cavities and hydrophobic domains that serve as reservoirs for payload encapsulation and allow environment-sensitive disassembly. Amphiphilic block copolymers, such as PMAO-PEG copolymers, have been utilized to template the assembly of UCNPs and encapsulate hydrophobic agents, including photosensitizers (e.g., ZnPc) or chemotherapeutics (e.g., DOX) [45]. These composite nanoclusters respond to external cues such as pH, light, or redox species. For instance, a PLGA-encapsulated UCNPs-DOX system exhibited pH-sensitive degradation and triggered DOX release in acidic tumor microenvironments while providing simultaneous fluorescence and MRI contrast due to Gd^3+^ doping [46]. Moreover, light-responsive systems based on UCNPs/polymer hybrids have shown excellent precision. One notable example involves UCNPs combined with photoreactive polymers that undergo structural rearrangement upon UV emission, indirectly activated via NIR-to-UV upconversion. This enables on-demand chemotherapy through the spatiotemporally controlled release of DOX and concurrent imaging (Figure 11a–b) [66]. In another design, ROS-sensitive polymer matrices surrounding UCNPs allow PDT to induce polymer degradation and facilitate drug release, resulting in synergistic chemo-PDT outcomes (Figure 11c–d) [65]. Zhu et al. introduced a multifunctional nanoplatform that integrates light-activated membrane disruption with redox-responsive drug release for enhanced intracellular delivery (Figure 11e–f) [110]. The system utilizes polymeric vesicles composed of disulfide-linked prodrug polymers, which co-encapsulate UCNPs and a photosensitizer (chlorin e6, Ce6). Under NIR excitation, UCNPs convert the input light to shorter wavelengths, activating Ce6 to generate reactive oxygen species that facilitate endosomal escape via photochemical internalization. Simultaneously, the elevated intracellular glutathione levels in tumor cells reduce the disulfide linkages within the polymer backbone, triggering localized release of the anticancer agent doxorubicin. This dual-responsive design enables both spatially controlled phototriggered activation and intracellular redox-driven drug liberation. The vesicular constructs exhibit excellent colloidal stability, effective light-triggered ROS production, and potent therapeutic outcomes in cellular and animal models. This work presents a strategic framework for developing stimulus-responsive nanocarriers that provide precise spatiotemporal control over drug release, with strong potential for applications in targeted cancer therapy and minimized systemic toxicity.

### 3.4. Orthogonal Photoactivation for Programmable Therapy

**Figure 12:**
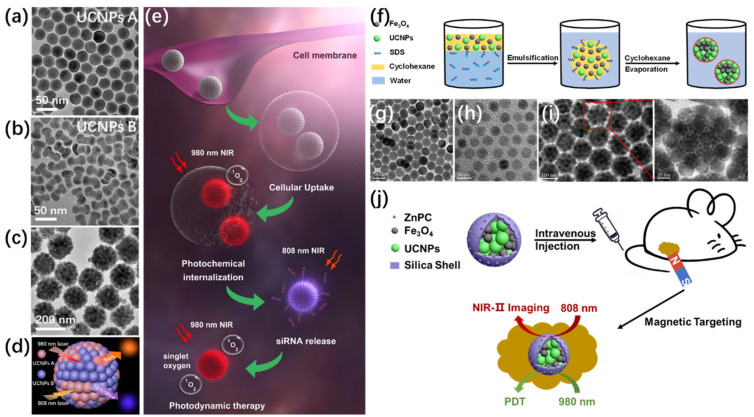
TEM images of two distinct UCNP types (**a**,**b**) and their assembled supercluster (**c**). (**d**) Diagram illustrating a UCNP cluster with dual-wavelength-responsive luminescence. (**e**) Schematic representation of wavelength-specific activation of photosensitizers and azobenzene-capped carriers for facilitating endosomal escape, siRNA release, and photodynamic therapy [116]. (**f**) Illustration of a microemulsion-assisted approach for co-assembling UCNPs and Fe_3_O_4_ into multifunctional superparticles. (**g**–**i**) TEM images of individual UCNPs, Fe_3_O_4_ nanoparticles, and the resulting MFSPs. (**j**) Schematic depiction of magnetically guided NIR-II imaging and photodynamic treatment in vivo [43].

One of the most promising directions in UCNP assembly lies in the development of orthogonally emissive platforms capable of wavelength-selective activation. These systems, known as orthogonal upconversion platforms, employ distinct lanthanide dopants that are independently excitable by different NIR wavelengths (e.g., 808 nm and 980 nm), allowing spatiotemporal control over multiple biological processes [23,111,112,113,114,115]. Using modular assembly approaches, researchers have constructed UCNP clusters in which distinct emission channels can be selectively triggered to perform separate functions, e.g., one for real-time imaging and another for drug release. For instance, a UCNP multifunctional supercluster coated with mesoporous silica was functionalized with azobenzene-based gates and loaded with chemotherapeutics (Figure 12a–e) [116]. The gates were cleaved upon UV emission under 808 nm NIR irradiation, releasing the drug, while 980 nm excitation simultaneously allowed for real-time imaging. This strategy prevents premature release and enables sequential therapeutic events such as photochemical internalization, gene silencing, and photodynamic therapy. Further optimization of cluster size (ideally 100–200 nm) ensures biological compatibility by avoiding renal clearance and enhancing tumor penetration. These superstructures exhibit high colloidal stability and robust performance over extended periods in physiological environments, demonstrating their viability as programmable theranostic platforms. Zhang’s group employed a microemulsion-assisted strategy to construct multifunctional UCNP/Fe_3_O_4_ hybrid superparticles, integrating magnetic targeting, PDT, and NIR-II imaging. This platform enables NIR-II imaging-guided PDT with real-time imaging capabilities (Figure 12f–j) [43].

## 4. Challenges and Future Perspectives

Despite the remarkable progress, several challenges remain to be addressed in the design and deployment of self-assembled UCNP composites. An essential consideration for UCNP-based nanocomposites is their colloidal and structural stability, which directly determines reproducibility, reliability, and long-term performance under realistic conditions. Colloidal stability is frequently challenged by nanoparticle aggregation in physiological ionic environments, fluctuations in pH, and nonspecific protein adsorption, whereas structural stability depends on the robustness of the assembly strategy, the interfacial binding strength among components, and the resistance to environmental stresses such as elevated temperature or irradiation. Surface chemistry is a pivotal factor governing the assembly behavior and overall functionality of UCNP-based nanocomposites. The choice and density of surface ligands critically influence interparticle spacing and assembly geometry, thereby dictating interfacial electronic coupling and photophysical outcomes. For example, short-chain or weakly binding ligands can minimize steric hindrance and promote compact assemblies, enhancing energy transfer efficiency but simultaneously increasing the risk of concentration quenching. In contrast, bulky or polymeric ligands expand interparticle spacing and improve colloidal stability, often at the cost of reduced photoluminescence efficiency. Functional moieties such as carboxyl, amine, or thiol groups introduce specific binding sites for covalent coupling or biorecognition-mediated assembly, enabling the design of tailored hybrid architectures for biosensing and therapeutic applications. Furthermore, advanced surface-engineering strategies such as PEGylation or zwitterionic modification not only stabilize colloidal dispersions under physiological conditions but also enhance biocompatibility and minimize nonspecific protein adsorption. Moreover, multifunctionalization—while desirable—introduces cross-reactivity and potential signal interference. Future research should explore modular and orthogonal functionalization methods, allowing independent control over multiple assembly domains. Stimuli-responsive assemblies that can be activated or disassembled by light, pH, or enzymes will enable intelligent systems for on-demand therapy, sensing, or release. Integration with microfluidic platforms may offer precise control over assembly conditions and throughput. Recent advances have also highlighted the potential of data-driven approaches, including machine learning and inverse design, for optimizing self-assembly parameters [117,118,119,120]. These strategies enable the prediction of optimal conditions for particle organization, interfacial stability, and optical performance, thereby reducing reliance on empirical trial-and-error. By coupling computational predictions with experimental validation, machine learning is emerging as a powerful tool to accelerate the discovery and rational design of UCNP-based nanocomposites. Artificial intelligence (AI)-driven design, predictive modeling, and data-informed synthesis optimization represent promising tools to accelerate material discovery. Additionally, regulatory and toxicity assessments will be crucial to ensure the safe translation of these advanced nanomaterials into biomedical and environmental applications. The translation of UCNP-based nanocomposites into clinical and technological use is further constrained by difficulties in large-scale and cost-effective synthesis, batch-to-batch reproducibility, surface modification to achieve biocompatibility, and compliance with regulatory standards. Addressing these challenges will require interdisciplinary efforts that integrate materials design, advanced surface engineering, scalable processing platforms (e.g., microfluidics, continuous-flow assembly), and standardized evaluation protocols. Future research should therefore focus not only on optimizing luminescent efficiency, but also on ensuring stability, reproducibility, and translational feasibility, which are indispensable for moving UCNP-based nanocomposites from laboratory demonstrations toward real-world biomedical and technological applications.

## 5. Conclusions

Self-assembly provides a versatile and modular route for engineering UCNP-based nanocomposites with tunable architecture and multifunctionality compared with traditional epitaxial growth (Table 2). By exploiting a range of electrostatic interactions, hydrophobic interactions, covalent coupling, and specific biorecognition, researchers have successfully constructed hybrid structures with enhanced optical, chemical, and biological performance. These systems are opening new frontiers in bioimaging, sensing, therapy, catalysis, and secure information technologies. Continued innovation in surface chemistry, stimuli-responsiveness, and assembly techniques, along with the integration of AI and microfabrication strategies, will undoubtedly shape the next generation of UCNPs nanotechnology.

## Figures and Tables

**Table 1:** Comparative overview of different self-assembly routes for UCNP-based nanocomposites.

Assembly Route	Operating Conditions	Inter-Particle Spacing/Structure	Optical Outcomes	Colloidal Stability	Biocompatibility
Electrostatic interactions	Highly dependent on pH, ionic strength, and surface charge	Tunable; typically a few nm; often flexible	Moderate quantum yield (QY); possible FRET enhancement due to close spacing	Sensitive to salt concentration; limited in physiological media	Moderate; surface charge may induce cytotoxicity
Hydrophobic interactions	Requires nonpolar solvents, amphiphiles, or surfactants	Compact, dense packing; anisotropic possible	Can enhance energy transfer via close packing; moderate photoluminescence (PL) control	Poor in aqueous media without further modification	Limited unless further coated; not inherently biocompatible
Covalent coupling	Requires functional ligands with reactive groups	Strong, permanent linkages; rigid structures	Stable PL efficiency; minimal quenching; better reproducibility	High stability due to irreversible bonding	Tunable with linkers; it depends on the chemistry used
Biorecognition-mediated assembly	Mild aqueous conditions; relies on biomolecular interactions	Highly specific, programmable, nm-scale precision	High control over FRET; precise PL modulation; multifunctional outputs	High if biomolecular linkers stabilized	High; especially with DNA, peptides, antibodies

**Table 2:** Side-by-side comparison of epitaxial growth and self-assembly approaches for UCNP-based nanocomposites.

Aspect	Epitaxial Growth	Self-Assembly
Interface	Atomically coherent, lattice-matched epitaxial interfaces; defect suppression	Noncovalent/covalent linkages; non-crystallographic, often flexible or reversible
Structure control	High precision at the atomic level; uniform shells and heterostructures	Versatile architectures with tunable interparticle spacing
Quantum yield	Typically high in optimized core@shell systems	Moderate, but enables enhanced functionalities (e.g., FRET, multi-modal imaging, catalysis)
Photostability	Excellent; strong suppression of surface quenching	Variable; depends on linker stability and environmental conditions
Scalability	Limited; requires high temperature, precise conditions, and complex synthesis	More scalable; solution-based, mild conditions, but reproducibility may vary with the environment
Biocompatibility	Requires surface modification (e.g., PEGylation) for aqueous/biological applications	Readily tunable via biomolecules, polymers, or responsive ligands

## Data Availability

Data sharing is not applicable.
